# Low unspliced cell-associated HIV RNA in early treated adolescents living with HIV on long suppressive ART

**DOI:** 10.3389/fimmu.2024.1334236

**Published:** 2024-02-20

**Authors:** Kathleen Gärtner, Sara Domínguez-Rodríguez, Judith Heaney, Triantafylia Gkouleli, Paul Grant, Karim Dorgham, Delphine Sauce, Cathia Soulie, Eloise J. Busby, Denise M. O’Sullivan, Moira Spyer, Johannes C. Botha, Maria Angeles Muñoz-Fernandez, Alfredo Tagarro, Nicola Cotugno, Jim F. Huggett, Nigel Klein, Paolo Palma, Pablo Rojo Conejo, Caroline Foster, Carlo Giaquinto, Paolo Rossi, Deborah Persaud, Anita De Rossi, Anne-Geneviève Marcelin, Eleni Nastouli

**Affiliations:** ^1^Great Ormond Street Institute of Child Health, University College London, London, United Kingdom; ^2^Fundación de Investigación Biomédica Hospital 12 de Octubre, Instituto de Investigación 12 de Octubre (imas12), Madrid, Spain; ^3^Universidad Europea de Madrid, Madrid, Spain; ^4^Advanced Pathogen Diagnostic Unit, University College London Hospitals (UCLH), London, United Kingdom; ^5^Haematology Department, Great Ormond Street Hospital for Children NHS Trust, London, United Kingdom; ^6^Sorbonne Université, Inserm, Centre d’Immunologie et des Maladies Infectieuses (CIMI-Paris), Paris, France; ^7^Sorbonne Université, Institut Pierre Louis d’Epidémiologie et de Santé Publique, Inserm, AP HP, Hôpitaux Universtaires Pitié Salpêtrière – Charles Foix, Laboratoire de Virologie, Paris, France; ^8^National Measurement Laboratory (NML), LGC Group, Teddington, United Kingdom; ^9^Clinical Immunology and Vaccinology Unit, Bambino Gesù Children's Hospital, Istituto di RicerCa a Carattere Scientifico (IRCCS), Rome, Italy; ^10^Laboratorio InmunoBiología Molecular, Hospital General Universitario Gregorio Marañón, Madrid, Spain; ^11^Department of Pediatrics, Infanta Sofía University Hospital, Fundación para la Investigación Biomédica e Innovación Hospital Universitario Infanta Sofía y Hospital del Henares (FIIB HUIS HHEN), Madrid, Spain; ^12^Academic Department of Pediatrics, Research Unit in Congenital and Perinatal Infections, Bambino Gesu Children’s Hospital, Rome, Italy; ^13^Department of Systems Medicine, University of Rome "Tor Vergata", Rome, Italy; ^14^School of Biosciences & Medicine, University of Surrey, Guildford, United Kingdom; ^15^Department of Paediatrics, Imperial College Healthcare NHS Trust, London, United Kingdom; ^16^Department of Surgery, Oncology and Gastroenterology (DiSCOG), University of Padova, Padova, Italy; ^17^Johns Hopkins University School of Medicine, Baltimore, MD, United States

**Keywords:** HIV-1, adolescents, early treated, suppressed, cell-associated RNA, cell-associated DNA, cytokines

## Abstract

**Introduction:**

Initiation of antiretroviral treatment (ART) in patients early after HIV-infection and long-term suppression leads to low or undetectable levels of HIV RNA and cell-associated (CA) HIV DNA and RNA. Both CA-DNA and CA-RNA, overestimate the size of the HIV reservoir but CA-RNA as well as p24/cell-free viral RNA can be indicators of residual viral replication. This study describes HIV RNA amounts and levels of cytokines/soluble markers in 40 well-suppressed adolescents who initiated ART early in life and investigated which viral markers may be informative as endpoints in cure clinical trials within this population.

**Methods:**

Forty adolescents perinatally infected with HIV on suppressive ART for >5 years were enrolled in the CARMA study. HIV DNA and total or unspliced CA-RNA in PBMCs were analyzed by qPCR/RT-qPCR and dPCR/RT-dPCR. Cell-free HIV was determined using an ultrasensitive viral load (US-VL) assay. Plasma markers and p24 were analyzed by digital ELISA and correlations between total and unspliced HIV RNA and clinical markers, including age at ART, Western Blot score, levels of cytokines/inflammation markers or HIV CA-DNA, were tested.

**Results:**

CA-RNA was detected in two thirds of the participants and was comparable in RT-qPCR and RT-dPCR. Adolescents with undetectable CA-RNA showed significantly lower HIV DNA compared to individuals with detectable CA-RNA. Undetectable unspliced CA-RNA was positively associated with age at ART initiation and Western Blot score. We found that a higher concentration of TNF-α was predictive of higher CA-DNA and CA-RNA. Other clinical characteristics like US-VL, time to suppression, or percent CD4+ T-lymphocytes were not predictive of the CA-RNA in this cross-sectional study.

**Conclusions:**

Low CA-DNA after long-term suppressive ART is associated with lower CA-RNA, in concordance with other reports. Patients with low CA-RNA levels in combination with low CA-DNA and low Western Blot scores should be further investigated to characterize candidates for treatment interruption trials. Unspliced CA-RNA warrants further investigation as a marker that can be prioritized in paediatric clinical trials where the sample volume can be a significant limitation.

## Introduction

International guidelines advise the immediate start of antiretroviral treatment (ART) in all HIV positive children and adults regardless of their plasma viral load (VL) status or CD4+ T-lymphocyte count (1). Early initiation of ART in HIV infection is correlated with decreased mortality and limited reservoir in long-term successful treatment (2–4). To ensure sustained HIV suppression ART must be taken continuously, meaning life-long for perinatally infected children, to prevent viral rebound, which typically occurs within 3-4 weeks after ART cessation (5, 6). There have been several reports where people living with HIV (PLWH) achieved viral control after ART cessation (termed post-treatment control, PTC), including the VISCONTI cohort (mean of 10 years to date) (7), the Mississippi baby (suppressed for 27 months before viral rebound) (8), and other subsequent studies (9, 10). However, markers and mechanisms that could predict PTC are not yet well understood and characterized. Early treated, well-suppressed children are expected to be a priority in clinical trials that include disease-modifying agents and analytical treatment interruption (ATI). Despite significant advances in characterizing the HIV reservoir in early treated children, better stratification of appropriate virological predictive markers of viral rebound post ATI is needed.

Different studies show a negative correlation between total HIV DNA before ATI and time to viral rebound (5, 6), however, cell-associated (CA) RNA has been suggested as a better predictive marker (11). Few studies exist about the levels of CA-RNA and their association with viral rebound after ATI (12). Undetectable plasma HIV RNA, i.e. below detection limit of CE-marked commercial assays, remains the gold standard and widely adopted marker for efficient viral suppression in patients on ART. However, HIV CA-RNA in PBMCs is frequently detected even in well suppressed individuals (13, 14) and is lower when ART is initiated early after infection (15).

During the HIV replication cycle different forms of viral RNA are expressed in a specific pattern (multiply or singly spliced during early replication, unspliced during late replication), which are translated into viral proteins and subsequently lead to packaging of full-length genomic (unspliced) viral RNA into HIV particles during virus assembly, followed by the release of infectious viruses. All different RNA forms contribute to the HIV CA-RNA pool (16). The LTR region of the HIV genome is present in all HIV CA-RNA species and can be used to measure total HIV CA-RNA, whereas unspliced HIV RNA is the only form that contains the gag/pol region and its detection indicates the presence of full-length genomic HIV RNA (17). Differential analysis of spliced and unspliced forms in combination with ultrasensitive VL measurements may determine whether active expression of viral proteins or residual viral replication in ART-suppressed HIV+ individuals could occur.

Furthermore, mRNA could trigger innate immune responses through nucleic acid sensing or the synthesis of HIV proteins from viral mRNAs could activate adaptive immune responses and might be the driver of persistent immune activation (18), as commonly observed [i.e. elevated levels of pro-inflammatory cytokines compared to healthy individuals (19, 20)]. Even after long-term suppressive ART these cytokine profiles do not normalize (21). The association between CA-RNA and cytokines in HIV infection is not well characterized. It is important to understand the levels of immune activation in individuals with detectable CA-RNA, indicating HIV protein expression, compared to those with undetectable CA-RNA.

This study aimed to characterize the CA-RNA in the CARMA cohort of very early treated suppressed adolescents (40 perinatally HIV-infected children, VL <50 copies/mL for a minimum of 5 years) by measuring both, CA-DNA and CA-RNA with different methods in view of their potential use in ATI trials. We further investigated viral and immune correlates, such as residual plasma VL and cytokine expression levels in plasma, as markers of potential ongoing viral expression driving immune activation in these young people.

## Methods

### Study participants

Forty children perinatally infected with HIV, who started ART within 2 years of age, were recruited into the CARMA cohort from 7 European centers of the EPIICAL consortium (22). Inclusion criteria were suppressed VL (<50 c/mL) for >5 years with maximum one blip (50-399 c/mL) or spike (400-999 c/mL) per year (**Supplementary Tables S1**, **S2**).

### Nucleic acid extraction from PBMCs and DNaseI treatment

PBMCs were purified from EDTA blood using Lymphoprep (Stemcell Technologies, Cambridge, UK) and nucleic acids were extracted as previously described with the QIAsymphony platform and the DSP virus/pathogen Mini kit (Qiagen, 22). For RNA analysis total nucleic acids were treated with DNaseI by adding 0.5µL DNaseI (2000U/ml, NEB) and 1µL 10x DNaseI buffer (NEB) to 10µL of extract followed by incubation for 10min at 37°C and 10min inactivation of the enzyme at 72°C.

### qPCR

**Figure 1:**
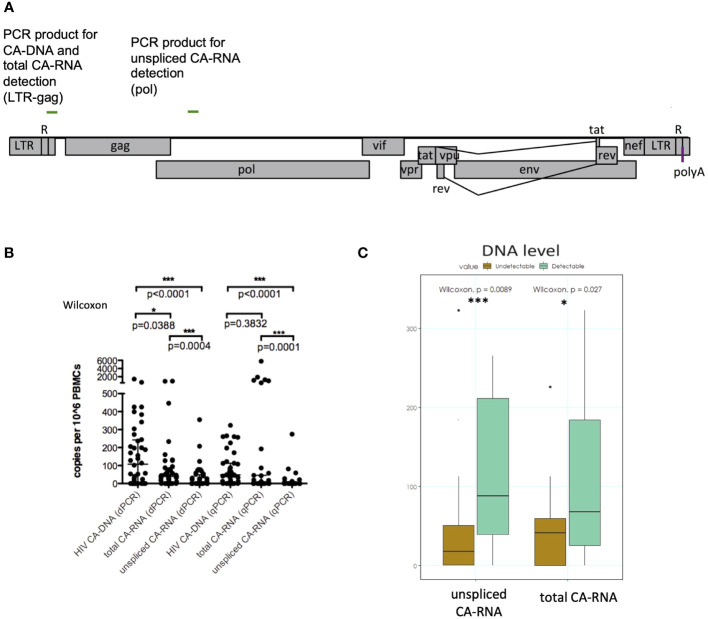
Analysis of HIV-1 cell-associated nucleic acids in children on ART; **(A)** LTR- and pol-primer/probe binding sites in the HIV-1 genome; **(B)** Detection of HIV-1 DNA, total and unspliced CA-RNA in PBMCs; **(C)** Level of CA-DNA in children with undetectable (brown column) and detectable (green columns) total and unspliced CA-RNA; Wilcoxon matched-pairs signed rank test *p<0.05, ***p<0.01.

CA-DNA was quantified with a PDH/HIV LTR-gag duplex assay as published [**Table 1**, **Figure 1A** (23)], using the QuantiTect Multiplex PCR Master Mix (Qiagen, Cat-No 204543, with ROX dye) and an HIV standard (8E5 cell extract with known copy numbers, 8E5 [derivative of CEM] ATCC CRL-3617). The assay was designed to detect a broad range of HIV subtypes (BREATHER trial, 24) and has also reliably been tested in a comparative study using subtypes A, B and C (25). For each 50µL reaction 10µL of nucleic acid extract was used. Thermocycling conditions were 15 min at 95°C followed by 45 cycles of 94°C for 60sec and 60°C for 60sec (see **Supplementary Table S3** for MIQE). The serially diluted 8E5 extract with known copy numbers (determined by dPCR) provided standard curves for PDH- and HIV-DNA. Analysis was done with the Applied Biosystems SDS v1.4 analysis software. Quantification of the PDH gene, present as two copies per diploid genome, was used to estimate the total number of cell equivalents in each amplification reaction. HIV DNA was quantified and displayed as copies per 10^6^ PBMCs (c/10^6^ PBMCs).

**Table 1:** Primers and probes used for detection of HIV CA-RNA and CA-DNA.

Primer name	Sequence	Reference
polFWDpg	TGTACCAGTAAAATTAAAGCCAGGAA	Detection of unspliced CA-RNA (24)
polREVpg	TATGGRTTTTCAGGCCCAATT	
HIVpol PROBEpg	FAM-TGGATGGBCCAARRGTYAAACARTGGCCATT-TAM	
HIV1LTRTaq1	GCCTCAATAAAGCTTGCCTTGA	detection of CA-DNA and total CA-RNA (23)
HIV1LTRTaq2	GGCGCCACTGCTAGAGATTTT	
HIV LTR Probe	FAM-TGTGACTCTGGTAACTAGAGATCCCTCAGAC-TAM	
PDH1	TGAAAGTTATACAAAATTGAGGTCACTGTT	Reference gene for CA-DNA assay (23)
PDH2	TCCACAGCCCTCGACTAACC	
PDH Joe/TAMRA	JOE-CCCCCAGATACACTTAAGGGATCAACTCTTAATTGT-TAM	
PrimePCR Probe Assay : IPO8, human, Cy5-labeled	Assay ID: qHsaCEP0057620	reference gene for CA-RNA assay (Bio-Rad)
PrimePCR Probe Assay : TBP, human, Hex-labeled, Bio-Rad	Assay ID: qHsaCIP0036255	reference gene for CA-RNA assay (Bio-Rad)

Total HIV CA-RNA was detected with the LTR-gag primers/probe [spliced plus unspliced, **Table 1** (23)] and unspliced RNA with pol primers/probe [**Table 1** (24)], using 10µL of DNaseI-treated extract in a 20µl one-step quantitative reverse transcription PCR reaction (RT-qPCR) with TaqMan Fast Virus master mix (Thermo Fisher, Cat-No 4444427). Human reference genes IPO8 (PrimePCR Probe Assay : IPO8, human, Cy5-labeled, Bio-Rad) and TBP (PrimePCR Probe Assay : TBP, human, Hex-labeled, Bio-Rad) were used in a triplex reaction. Cycling conditions were 5min at 50°C, 20sec at 95°C followed by 45 cycles of 15sec at 94°C and 90sec at 60°C. A synthetic HIV RNA transcript with known copy number (determined by RT-dPCR) was included as standard curve. Results were analyzed with CFX Maestro Software (Bio-Rad). TBP and IPO8, were used to ensure consistent sample quantification. HIV copy numbers were calculated per 10^6^ PBMCs relative to sample input in the reaction (10µl of 200µl extract from 5x10^6^ PBMCs).

Calculation of HIV copy numbers was based on the DNA- or RNA-standard curve, where copy numbers were defined through the according Ct values. In successful experiments standard curves showed an efficiency R^2^≥0.95.

### dPCR

The digital droplet PCR (dPCR) to detect HIV proviral DNA was performed with ddPCR Supermix for Probes (No dUTP, Bio-Rad, Cat-No 186-3023) in a 22µL multiplex reaction (sample input 8.8µl, see **Supplementary Table S4** for dMIQE), using HIV LTR and PDH primers/probe as reference gene [**Table 1** (23)]. Droplet formation was performed on a Manual Droplet Generator (Bio-Rad) and the plate sealed with a plate sealer before running the PCR in a thermocycler. Cycling conditions were 10min at 95°C (ramp rate 2°C/sec) followed by 40 cycles of 30sec at 94°C and 1min at 60°C (ramp rate 2°C/sec), and a final denaturation step for 10min at 98°C. Analysis of the droplets was done with the QX100 droplet reader and the QuantaSoft software, version 1.7.4 (Bio-Rad). HIV DNA was quantified and displayed as c/10^6^ PBMCs.

For quantification of CA-RNA 5µL of DNaseI-treated extract were analyzed for total HIV RNA and unspliced RNA (**Table 1** (23)). A one-step reverse transcription dPCR using the One-Step RT ddPCR Supermix (Bio-Rad, Cat-No 1864021) was performed in a 22µL multiplex reaction, using TBP-1 (PrimePCR Probe Assay : TBP, human, Hex-labeled, Bio-Rad) as reference gene. After droplet formation with the Automated Droplet Generator (Bio-Rad) the PCR was performed for 60min at 50°C, 10min at 95°C followed by 40 cycles of 30sec at 95°C and 1min at 56°C and a final denaturation step for 10min at 98°C. Analysis of the droplets was done as before. TBP was used to ensure consistent sample quantification and HIV copy numbers were calculated per 10^6^ PBMCs relative to sample input in the reaction (10µl of 200µl extract from 5x10^6^ PBMCs).

### Ultrasensitive VL assay

HIV virions were pelleted from 8mL of plasma by centrifugation at 21,000x g for 2 hours at 4°C (26). Supernatants were removed and virus pellets resuspended in 750µL human HIV negative plasma before VL determination (pol and LTR) on the Hologic Panther utilizing the Aptima^®^ HIV-1 Quant Assay. The limit of detection (LOD) was 1.05 c/mL, as determined using the 3rd HIV-1 WHO International Standard (subtype B, NIBSC code: 10/152).

### Western blot score assay

The Western blot score in plasma was determined using the Abbott Architect HIV Ag/Ab Combo Assay (fourth generation) according to the manufacturer’s protocol. A score was added up for each sample depending on the number of HIV proteins the plasma was reactive to [between 0-10 (27)].

### Digital ELISA

The Simoa™ (single molecule array) HD-1 analyzer (Quanterix, Lexington, MA, USA) was used for ultrasensitive bead-based immunodetection (digital ELISA) with single-plex assays to detect HIV-1 p24 (Quanterix, Cat-No 102215), soluble (s)PD-L1 (Quanterix, Cat-No 102648) and sPD-1 (Quanterix, Cat-No 102929), and a triplex assay to detect IL-10, IL-6 and TNF-α (Quanterix, Cat-No 101160) according to the manufacturer’s instructions. At low concentration, the percentage of bead-containing wells with a positive signal is proportional to the amount of analyte present in the sample (digital measurement). At higher concentration, most of the bead-containing wells have one or more labeled analytes, the total fluorescence signal is proportional to the amount of analyte present in the sample (analog measurement). Calibrators were run in duplicate and fit with a 4-parameter logistic (4PL) regression, with 1/y^2^ weighting.

Levels of IP-10 (Invitrogen, Cat-No EPX01A-10284-901), MCP-1 (Invitrogen, Cat-No EXP01B-10281-901) and VCAM-1 (Invitrogen, Cat-No EXP01A-20619-901) were assessed by the Procartaplex using ultrasensitive Luminex technology according to the manufacturer’s protocols.

### Statistical analysis

Univariable correlation analysis between CA-DNA and CA-RNA (**Figure 1B**) was performed with GraphPad Prism Version 5.0c. Wilcoxon matched-pairs signed rank test was used to compare data.

In order to describe the study population, data were displayed as a whole, and summarized by CA-RNA groups [detectable: >0copies (c); undetectable 0c]. For continuous variables, medians and interquartile ranges (IQR) were assessed and counts and percentages for categorical variables. Chi-squared was performed to compare between groups in case of categorical variables and Mann-Whitney-Wilcoxon when continuous. Hypothesis testing was carried out at the 5% significance level and p-values rounded to three decimal places.

The concordance between qPCR and dPCR to measure HIV CA-DNA was determined using the Bland-Altman test. HIV DNA from the same extraction was used in duplicates to compare the two methods.

To assess the association between CA-RNA and CA-DNA levels, a univariable and multivariable negative binomial regression model was applied adjusted for age at ART, baseline VL, and baseline percentage of CD4. Zero-inflation was tested using vuong test (28) and checking density distribution function. Variable selection was performed using backward stepwise AIC criterion. Additionally, a univariable and multivariable logistic regression was applied to test the association of undetectable CA-DNA (<10 c/mL) and undetectable total/unspliced CA-RNA (0c/mL).

Clinical and virologic predictors were compared between detectable and undetectable CA-RNA groups segregated by unspliced and total CA-RNA using Mann-Whitney-Wilcoxon test.

To assess the association between cytokines/soluble inflammation markers and CA-DNA levels, we performed ridge poisson regression selecting lambda values by a 5-fold cross-validation implemented in glmnet package (29). Relative variable importance was calculated to assess the prediction importance of each cytokine/inflammation marker included in the model. The data were adjusted for age at ART, baseline % CD4 and baseline VL.

## Results

In the CARMA cohort of 40 perinatally infected children total CA-RNA was undetectable in 22.5% and unspliced CA-RNA in 42.5% of the participants using either RT-qPCR or RT-dPCR (**Table 2**). Where CA-RNA could be detected it was very low and comparable between the two methods, with medians of 23 and 29 c/10^6^ PBMCs for total CA-RNA and medians of 19 and 18 c/10^6^ PBMCs for unspliced CA-RNA in RT-qPCR and RT-dPCR, respectively. Both methods showed high concordance in Bland-Altman testing, with agreement of 97.4% for total and 94.7% for unspliced RNA (**Supplementary Figure S1A**), suggesting they are equally suitable for HIV CA-RNA detection. Subsequent analyses were done with values obtained from RT-qPCR if not stated otherwise.

**Table 2:** Detection of CA-DNA and CA-RNA in 40 long-term suppressed, early treated CARMA participants.

	qPCR c/10^6^ PBMCs (CI)	dPCR c/10^6^ PBMCs (CI)	Negative with qPCR and dPCR	Positive with qPCR and dPCR	Positive only with qPCR	Positive only with dPCR
**CA-DNA**	81 (0.1-323)	174 (19–1420)	3/40	27/40	9/40	1/40
**total RNA**	23 (1-5789)	29 (11-857)	9/40	21/40	2/40	8/40
**US RNA**	19 (0.04-274)	18 (11-325)	17/40	14/40	5/40	4/40

CA-DNA – cell-associated DNA; US RNA – unspliced RNA.

Unspliced CA-RNA copy numbers were significantly lower compared to CA-DNA (**Figure 1B**). Total CA-RNA copy numbers were lower than the CA-DNA levels in both methods (dPCR and qPCR) but only reached significance with dPCR (p=0.0388). This could be due to the greater number of samples with higher than 1000 c/10^6^ PBMCs (**Figure 1B**) measured by RT-qPCR. Due to limited volume of samples, RT-qPCR and RT-dPCR could not be repeated to confirm these results. **Figure 1C** shows that participants with undetectable total or unspliced CA-RNA (brown columns) present a lower CA-DNA level, whereas participants with detectable CA-RNA (green columns) had higher CA-DNA. Total CA-RNA showed a correlation with CA-DNA levels (p=0.0016, **Supplementary Figure S1B**), whereas unspliced CA-RNA was not correlated with CA-DNA (p=0.2424, **Supplementary Figure S1B**).

**Figure 2:**
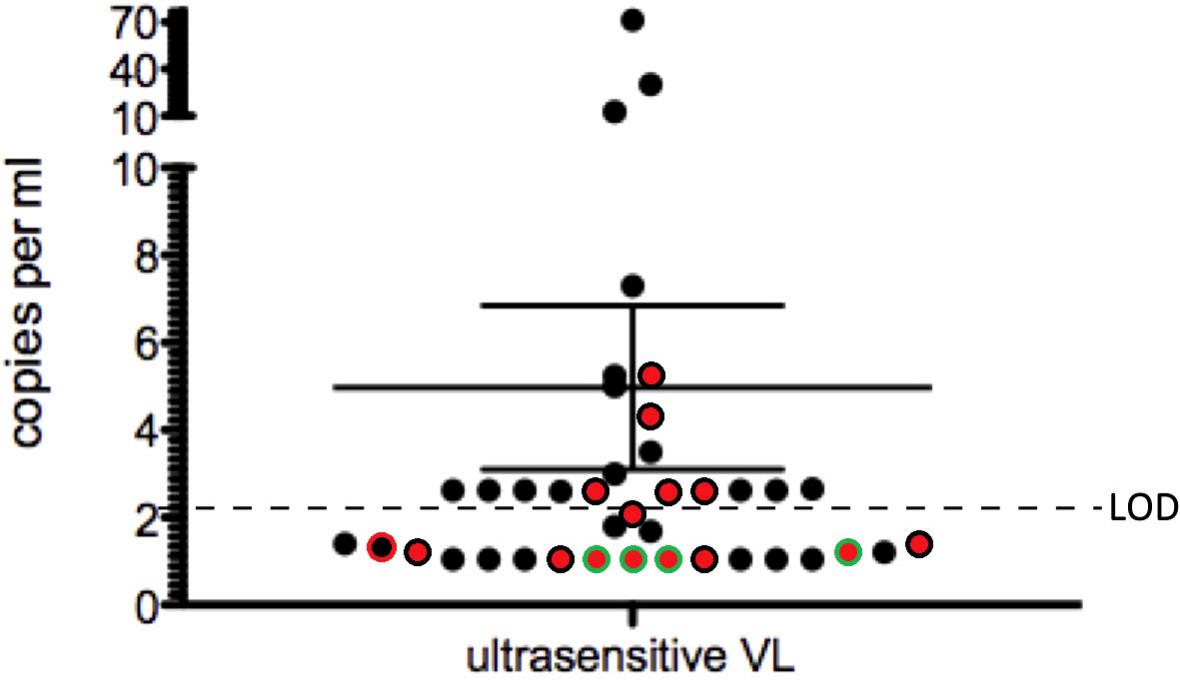
Detection of ultra-sensitive VL in CARMA participants. Samples with detectable CA-RNA plus CA-DNA are black, samples with negative CA-RNA are depicted with a green circle and samples with negative CA-DNA have a red center. Double-negative samples are red with a green circle. Shown is the mean of all samples with 95% confidence interval.

Since unspliced HIV CA-RNA was detected in PBMCs and therefore could be indicative of virus replication despite ART, we used an ultrasensitive VL assay (US-VL) to determine low-level cell-free virus in plasma. US-VL was below the limit of detection in 20 of the 40 participants and below 10 copies in 17 of the 20 positive samples (**Figure 2**). Only three samples showed values above 10 c/mL (12.6, 30.2, 71.4 c/mL). We did not find statistically significant correlations of US-VL with the CA-DNA level or with total and unspliced CA-RNA (data not shown), probably due to limited numbers of participants. Furthermore, HIV p24 protein in plasma was measured with a digital ELISA and mean values were near the limit of detection of the assay (data not shown). Together with the ultrasensitive viral load assay this confirms the well-suppressed state of the CARMA participants.

**Figure 3:**
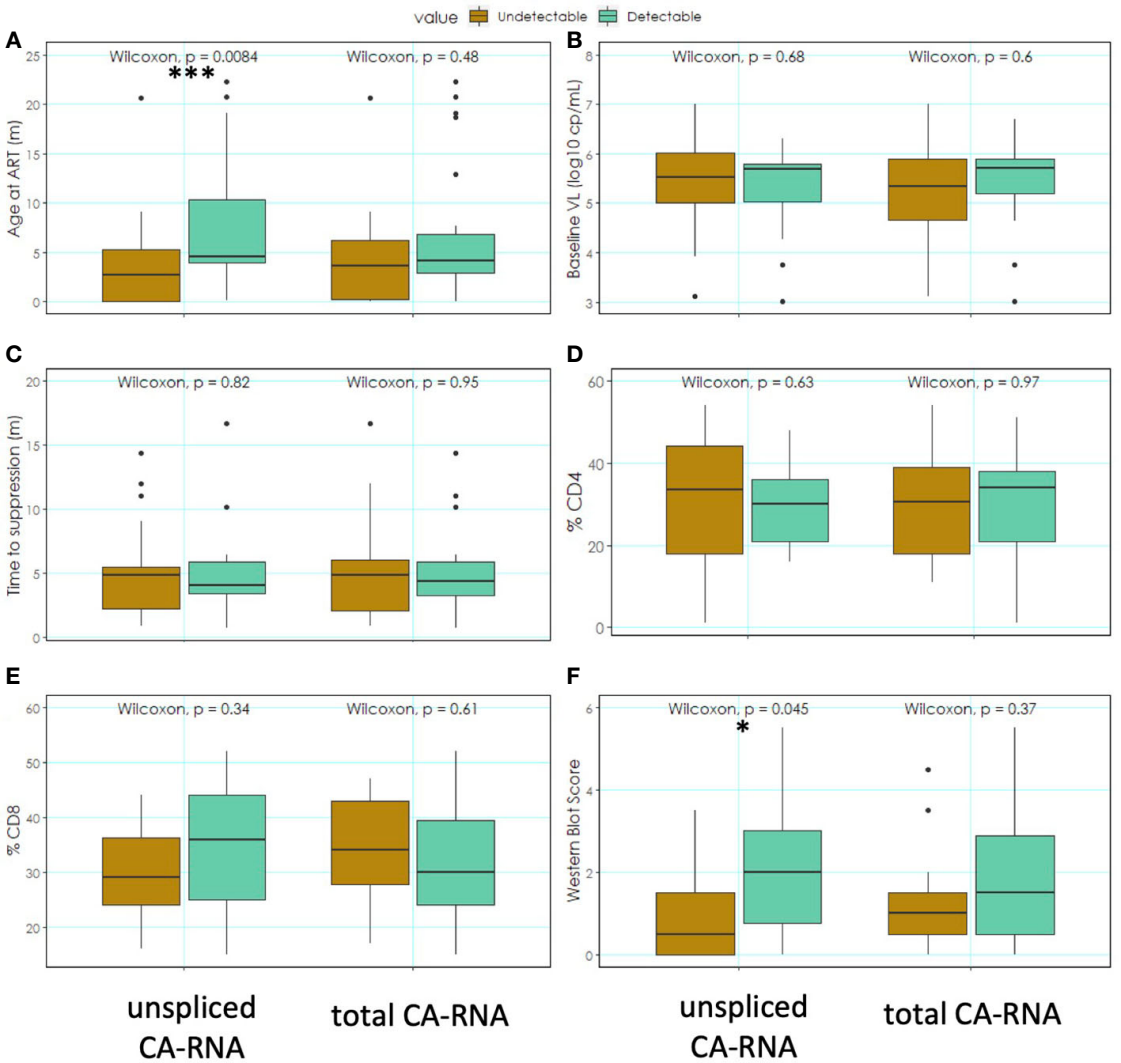
Clinical markers in groups of participants with detectable and undetectable CA-RNA; **(A)** Age at ART, **(B)** Baseline VL, **(C)** Time to suppression, **(D)** %CD4 T-lymphocytes, **(E)** %CD8 T-lymphocytes, **(F)** Western Blot Score. Shown are medians with 95% confidence interval; Wilcoxon matched-pairs signed rank test *p<0.05, ***p<0.01.

Other markers associated with the CA-RNA on ART could be age at ART initiation (**Figure 3A**), baseline VL (**Figure 3B**), time to suppression (**Figure 3C**), %CD4 (**Figure 3D**) and %CD8 (**Figure 3E**) T-lymphocytes at ART initiation or Western Blot Score [**Figure 3F**, **Supplementary Tables S1**, **S2** (22)]. Patients were categorized according to detectable (**Figure 3**, green columns) or undetectable (**Figure 3**, brown columns) total or unspliced RNA. The patient group with undetectable unspliced CA-RNA showed a significantly lower Western Blot Score (p=0.044, **Figure 3F**) and age at ART (p=0.008, **Figure 3A**) compared to individuals with detectable unspliced HIV CA-RNA (**Figure 3**; **Supplementary Tables S1**, **S2**). The other markers tested did not show significant difference between undetectable and detectable unspliced CA-RNA (**Figure 3**). For total CA-RNA no correlations with these markers were found.

It has been shown that persistent immune activation can be observed in HIV+ individuals despite ART (19, 20). The positive correlation of unspliced CA-RNA and Western Blot score in the ART-treated pediatric patients could indicate a low level of virus particle production and subsequent antibody production leading to a higher Western Blot score. This is unlikely since we show very good suppression in our cohort. However, protein expression from CA-RNA could contribute to persistent immune activation (i.e., altered cytokine levels compared to HIV uninfected individuals) and might influence antibody production through B-cell activation. We measured eight different cytokines and soluble inflammation markers in the plasma of the CARMA participants (**Table 3**) to determine associations between those and the total or unspliced CA-RNA. The participants were divided in two groups, detectable vs. undetectable CA-RNA.

**Table 3:** Detection of cytokines and inflammation markers in plasma by digital ELISA.

pg/mL	CARMA Mean (SD)	Healthy adult controls (SD)	p-value*
**sPD-L1**	67.8 (22.91) n=40		
**IL-10**	1.7 (3.3) n=40	0.463 (0.238) n = 29	p<0.0001
**IL-6**	1.59 (2.60) n=40	0.9727 (0.817) n =38	n.s.
**TNF- α**	3.06 (0.95) n=40	2.103 (0.700) n = 38	p<0.0001
**sPD-1**	434.95 (236.29) n=40		
**IP-10**	3.98 (4.92) n=40		
**MCP1**	4.46 (3.11) n=39		
**sVCAM-1**	42,169.48 (41,725.47) n=26		

*Unpaired t-test with Welch’s correction. p<0.05 is significant. n.s. – not significant.

There are a few studies of cytokine analysis in healthy children or adolescents, which showed similar values to our HIV+ cohort (30). Data for cytokines IL-10, TNF-α, and sPD-1 were available from healthy adults. Compared to these values the amounts of IL-10, TNF-α, and sPD-1 were increased in the CARMA cohort. When samples were divided into groups with detectable and undetectable CA-RNA, we could see a significantly lower amount of sVCAM-1 in the group with undetectable total CA-RNA compared to detectable total CA-RNA (p=0.012) but no other correlations were identified (**Supplementary Tables S1**, **S2**).

To determine associations between total or unspliced CA-RNA we did univariable and multivariable tests, adjusted for age at ART, baseline %CD4 and baseline VL (**Supplementary Tables S5**, **S6**). An association between total CA-RNA and sPD-1 (p=0.028), sPD-L1 (p=0.0001) and MCP-1 (p=0.013) in a multivariate analysis and total CA-RNA and MCP-1 (p=0.016) in univariate analysis was observed. For samples with detectable total CA-RNA one unit increase in MCP-1 was associated with a 33% decrease and one unit increase in PDL-1 is associated with a 7% increase of total CA-RNA. Unspliced CA-RNA did not show an association with any of the measured markers in the plasma.

**Figure 4:**
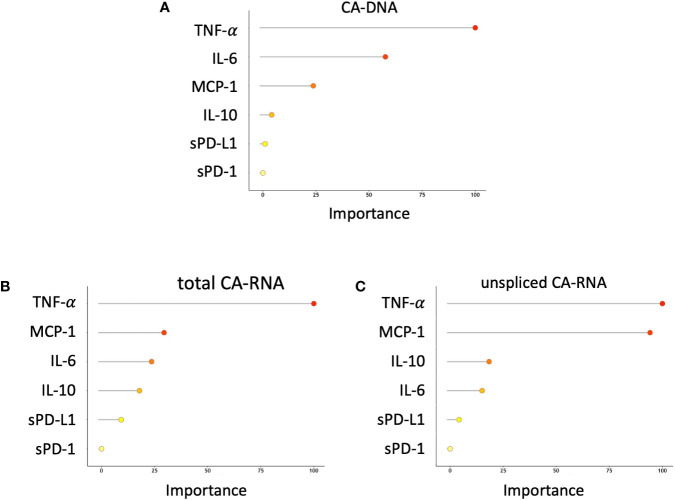
Importance of different cytokines and inflammation markers for prediction of increasing **(A)** HIV CA-DNA; **(B)** total CA-RNA; **(C)** unspliced CA-RNA.

We also tested a prediction model (using ridge poisson regression, see Methods) to evaluate the importance of different cytokines/inflammation markers for the different HIV nucleic acid forms in our participants. The most important markers for the prediction of higher amounts of CA-DNA and total or unspliced CA-RNA were TNF-α, MCP-1, IL-10, and IL-6 (**Figure 4**, adjusted for age at ART initiation, baseline VL and baseline %CD4).

## Discussion

To analyze the expression of different HIV CA-RNA species in PBMCs we used two methods, RT-qPCR and RT-dPCR, and could show that both methods generate comparable results when detecting low copy numbers of different CA-RNA forms. This is an important finding, because diagnostic laboratories are usually only equipped with either, a qPCR or a dPCR system. Despite high agreement between the two methods (Bland-Altman test, **Supplementary Figure S1**) there were three outliers with high amounts of total CA-RNA in RT-qPCR, which are not in agreement with the dPCR results (**Figure 1B**). Unfortunately, the analysis of these samples could not be repeated due to limited amounts of samples.

Studies have already shown that low levels of HIV CA-DNA and CA-RNA leads to longer times to viral rebound after treatment interruption (5, 6). In the present study we analyzed clinical and virological markers (**Table 3**), which could add important information for the identification of individuals who may potentially achieve PTC. Seven out of 40 CARMA participants did not show any detectable CA-RNA (neither total nor unspliced), the remainder had very low CA-RNA amounts. We could see that participants with undetectable CA-RNA (total and unspliced) showed lower CA-DNA, which is in agreement with a study by Hong et al. (31), who showed that lower unspliced CA-RNA is associated with lower CA-DNA in untreated or ART-treated patients. In the CARMA study, only total CA-RNA was correlated with CA-DNA, but not unpsliced, which might be due to the small number of participants. Combined with the extremely low amounts of plasma VLs detected, this confirms good suppression of HIV in this early ART-treated cohort with perinatal infection. Nevertheless, we could detect different CA-RNA species in PBMCs in the majority of our participants, indicating potential for residual HIV protein expression without particle production. Also, the detection of unspliced CA-RNA, presumed to be genomic HIV RNA, indicates the potential for residual viral replication in PBMCs in some participants. The absence of unspliced CA-RNA, on the other hand, could indicate the absence of replication competent viral sequences and longer time to viral rebound after treatment interruption. To analyze whether these unspliced, presumed genomic RNAs in PBMCs could be replication competent, sequencing of the proviral landscape and virus induction assays are currently analyzed in our laboratory. This could help to further characterize our cohort and highlight markers that are most informative in regard of the HIV reservoir and viral rebound after ATI.

In respect of the other clinical markers unspliced CA-RNA in PBMCs was positively associated with Western Blot score and age at ART. This profile of early treated children with lower Western Blot score and lower unspliced CA-RNA emerges in our study as an ideal one with regards to selecting patients for novel immunomodulatory therapies. We showed that these children have better preserved immune profile and our findings confirm less active virological markers ideal for immune modifying therapeutic vaccines. Unspliced CA-RNA warrants further investigation as a marker of the HIV reservoir and as a potential endpoint for clinical trials. To further deepen the understanding about relationships between CA-RNA and other clinical and virological markers, other forms of HIV CA-RNA could be analyzed. Tat or Rev mRNA (both multiply spliced) could be interesting candidates, because the expressed proteins play important roles in enhancement of viral replication and export of genomic HIV RNA into the cytoplasm, respectively.

Another important characteristic of HIV infection is persistent immune activation, which is indicated by elevated concentrations of inflammation markers despite ART. The levels of the pro-inflammatory cytokines IL-6, IL-10, and TNF-α as well as IP10, MCP1 and VCAM-1 were not increased in the CARMA participants, compared to healthy children of the same age range (30). However, it has been published that HIV+ children at younger age show an elevated immune activation compared to healthy controls (19, 32) or adults (33). These results could be confirmed by collecting an additional sample from the CARMA participants, however, this was not part of the study. Such cytokines/inflammation markers should be investigated as potential therapeutic targets to avoid immunosenescence, premature ageing or cardiovascular disease in PLWH and as possible markers for post-treatment HIV control.

A more direct analysis of isolated lymphocyte subsets, macrophages, or NK cells *ex vivo* could better elucidate the ability of those cells to produce cytokines when challenged with HIV. Our group previously described that early ART initiation is associated with lower activation status of natural killer cells and consequently lower cytokine production (34).

There are some limitations in our study. For the detection of very low cell-associated HIV RNA we used purified PBMCs whereas isolation of CD4+ T-lymphocytes, the main target cells for HIV, would increase the concentration of virus RNA detected per cell and negative values would be even more informative. But this is only feasible if sufficient blood can be drawn, which is difficult in pediatric patients, where sample volumes are limited. The detection of negative values of CA-RNA and plasma RNA in the CARMA cohort confirm the very good suppression in this group. Analysis of a subsequent sample from the CARMA participants would provide additional important information about sustained suppression of HIV CA-RNA expression and also indications about whether CA-RNA expression is consistent over time or is decreasing even further. Additionally, the assays used in this study are not able to distinguish between intact and non-intact sequences.

In conclusion, this study adds important findings to the field of perinatal HIV-infection in children with early ART initiation: (i) Quantification of CA-RNA and CA-DNA is reliably detected with both, qPCR and dPCR assays, demonstrating their suitability for use in clinical trials. (ii) Undetectable unspliced, but not total CA-RNA is associated with early ART-initiation and these individuals show lower CA-DNA levels. (iii) The detection of two different CA-RNA forms indicates some viral activity (protein expression or even particle production), however, it does not seem to have a great impact on the activation state of the immune system in well controlled adolescents.

Our results suggest that the absence of detectable CA-RNA combined with additional virological and clinical markers (low Western Blot Score, young age of ART initiation) may be useful in selecting individuals for ATI trials, and/or can be used as an endpoint for future clinical trials. Although ATIs pose the risk of viral rebound, the naïve profile of the immune system after early suppression of HIV replication could also lead to prolonged time to viral rebound and lessen the burden of drug toxicity, therefore, ethics in children cohorts need to be considered carefully (35). Our study could help to inform such decisions.

## Data availability statement

The raw data supporting the conclusions of this article will be made available by the authors, without undue reservation.

## Ethics statement

The studies involving humans were approved by ethics committees in each country. The studies were conducted in accordance with the local legislation and institutional requirements. Written informed consent for participation in this study was provided by the participants’ legal guardians/next of kin.

## Author contributions

KG: Conceptualization, Data curation, Formal analysis, Investigation, Methodology, Supervision, Validation, Writing – original draft, Writing – review & editing, Visualization. SD-R: Data curation, Formal analysis, Software, Writing – review & editing, Validation, Visualization. JHe: Data curation, Formal analysis, Investigation, Methodology, Writing – review & editing. TG: Data curation, Formal analysis, Investigation, Validation, Visualization, Writing – review & editing. PG: Data curation, Formal analysis, Investigation, Methodology, Validation, Writing – review & editing. KD: Data curation, Formal analysis, Investigation, Methodology, Visualization, Writing – review & editing, Validation. DS: Data curation, Formal analysis, Investigation, Methodology, Writing – review & editing, Validation. CS: Data curation, Formal analysis, Investigation, Methodology, Visualization, Writing – review & editing, Validation. EB: Methodology, Validation, Writing – review & editing, Data curation, Formal analysis, Investigation. DO: Data curation, Formal analysis, Investigation, Methodology, Validation, Writing – review & editing. MS: Conceptualization, Funding acquisition, Project administration, Supervision, Writing – review & editing. JB: Formal analysis, Methodology, Writing – review & editing. MM-F: Conceptualization, Resources, Supervision, Writing – review & editing. AT: Conceptualization, Funding acquisition, Project administration, Resources, Supervision, Writing – review & editing. NC: Data curation, Formal analysis, Investigation, Methodology, Writing – review & editing. JHu: Conceptualization, Formal analysis, Supervision, Writing – review & editing. NK: Conceptualization, Funding acquisition, Project administration, Resources, Supervision, Writing – review & editing. PP: Conceptualization, Funding acquisition, Project administration, Supervision, Writing – review & editing. PRC: Conceptualization, Funding acquisition, Project administration, Supervision, Writing – review & editing. CF: Conceptualization, Funding acquisition, Project administration, Supervision, Writing – review & editing. CG: Conceptualization, Funding acquisition, Project administration, Resources, Supervision, Writing – review & editing. PR: Conceptualization, Funding acquisition, Project administration, Resources, Supervision, Writing – review & editing. DP: Conceptualization, Writing – review & editing. AD: Conceptualization, Formal analysis, Project administration, Resources, Supervision, Writing – review & editing. A-GM: Conceptualization, Formal analysis, Funding acquisition, Supervision, Writing – review & editing. EN: Conceptualization, Formal analysis, Funding acquisition, Project administration, Supervision, Writing – original draft, Writing – review & editing.

## Supplementary material

Supplementary Figure 1 **(A)** Bland-Altman test of RT-qPCR and RT-dPCR for detection of HIV-1 total and unspliced CA-RNA (analyzed as copy numbers per reaction); Confidence interval depicted by the dashed lines. **(B)** Correlations between total and unspliced CA-RNA with CA-DNA; Two-tailed Pearson test.

Supplementary Table 1 Immunological and virological markers of CARMA participants with detectable or undetectable total CA-RNA.

Supplementary Table 2 Immunological and virological markers of CARMA participants with detectable or undetectable unspliced CA-RNA.

Supplementary Table 3 MIQE.

Supplementary Table 4 dMIQE.

Supplementary Table 5 Association of clinical markers and cytokines/inflammation markers with total CA-RNA.

Supplementary Table 6 Association of clinical markers and cytokines/inflammation markers with unspliced CA-RNA.
