# Supplementary figures and images for: Low unspliced cell-associated HIV RNA in early treated adolescents living with HIV on long suppressive ART
Source: Front Immunol. 2024 Feb 20;15:1334236. doi: 10.3389/fimmu.2024.1334236 (PMC10912947; doi:10.3389/fimmu.2024.1334236)

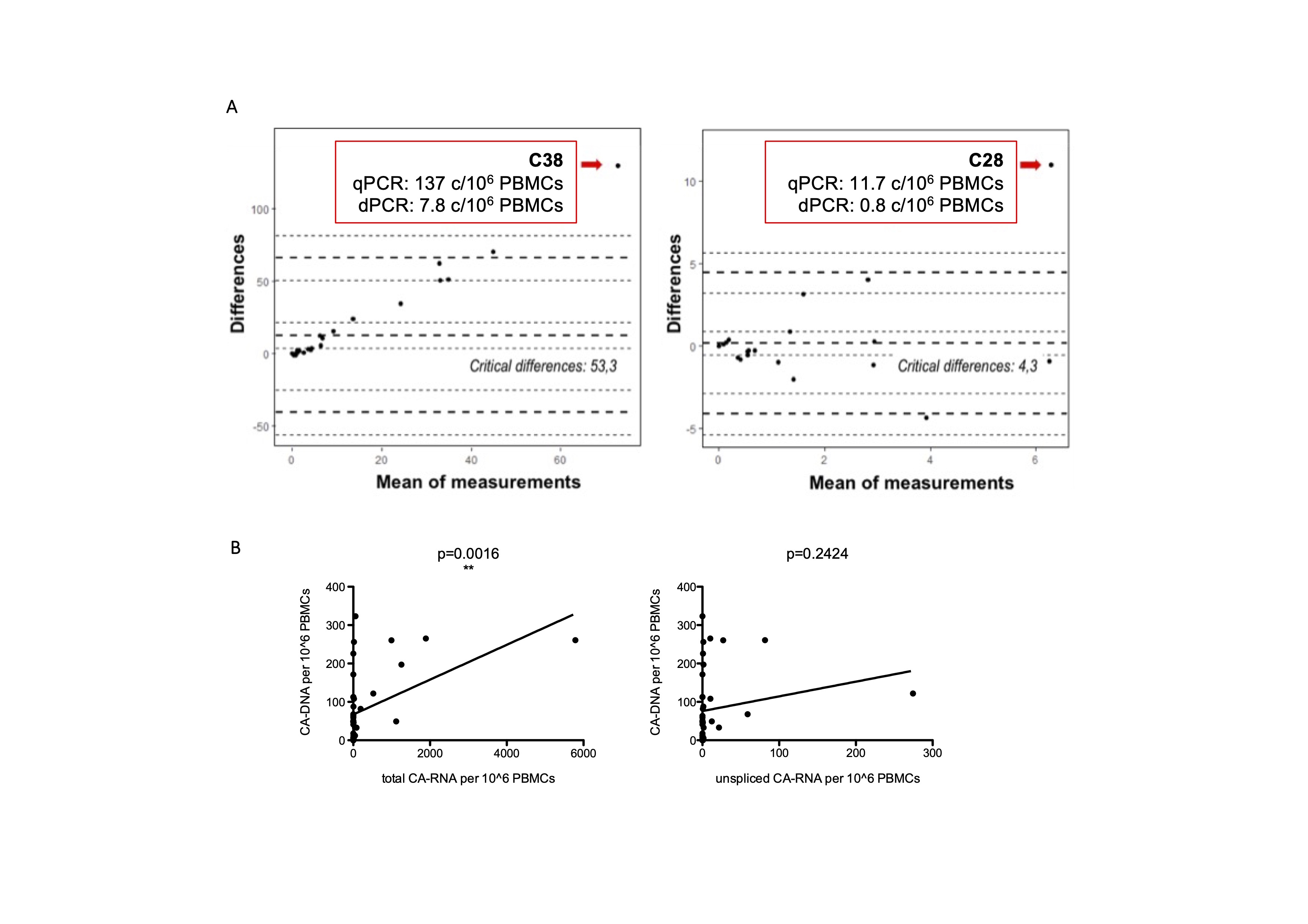

Supplement: Supplementary Figure 1 — (A) Bland-Altman test of RT-qPCR and RT-dPCR for detection of HIV-1 total and unspliced CA-RNA (analyzed as copy numbers per reaction); Confidence interval depicted by the dashed lines. (B) Correlations between total and unspliced CA-RNA with CA-DNA; Two-tailed Pearson test. [file Image_1.jpg]
