# Supplementary material for: Low unspliced cell-associated HIV RNA in early treated adolescents living with HIV on long suppressive ART
Source: Front Immunol. 2024 Feb 20;15:1334236. doi: 10.3389/fimmu.2024.1334236 (PMC10912947; doi:10.3389/fimmu.2024.1334236)
Supplement: Supplementary Table 1 — Immunological and virological markers of CARMA participants with detectable or undetectable total CA-RNA. [file Table_1.docx]

**Supplementary Table S1: Immunological and virological markers of CARMA participants with detectable or undetectable total CA-RNA**

|  | **Total** | **total CA-RNA >0cp/mL** | **total CA-RNA 0cp/mL** | **p-value** |
| --- | --- | --- | --- | --- |
|  | ***N=40*** | ***N=23*** | ***N=17*** |  |
| **Gender:** |  |  |  | **0.484** |
| **Male** | **13 (32.5%)** | **9 (39.1%)** | **4 (23.5%)** |  |
| **Female** | **27 (67.5%)** | **14 (60.9%)** | **13 (76.5%)** |  |
| **Age at ART (m)** | **4.08 [0.25;6.23]** | **4.13 [2.89;6.82]** | **3.61 [0.20;6.16]** | **0.467** |
| **Age at HIV diagnosis** | **4.17 [2.19;6.32]** | **4.20 [3.12;6.14]** | **4.03 [1.21;6.26]** | **0.547** |
| **Baseline Viral load (log10 cp/mL)** | **5.60 [4.98;5.93]** | **5.70 [5.19;5.90]** | **5.34 [4.66;5.90]** | **0.593** |
| **Time to suppression (m)** | **4.69 [2.52;6.26]** | **4.39 [3.26;5.85]** | **4.85 [2.26;8.36]** | **0.805** |
| **DNA amounts** | **48.3 [6.65;113]** | **67.7 [25.3;184]** | **41.1 [0.10;59.3]** | **0.026** |
| **Baseline CD4 count** | **31.0 [18.0;38.0]** | **34.0 [21.0;38.0]** | **30.5 [18.0;39.0]** | **0.955** |
| **Baseline % CD4** | **32.0 [25.0;40.0]** | **30.0 [24.0;39.5]** | **34.0 [27.8;43.0]** | **0.597** |
| **Baseline % CD8** | **4.69 [2.52;6.26]** | **4.39 [3.26;5.85]** | **4.85 [2.26;8.36]** | **0.805** |
| **AntiCMV IGG cat:** |  |  |  | **0.071** |
| **Negative** | **10 (25.6%)** | **3 (13.6%)** | **7 (41.2%)** |  |
| **Positive** | **29 (74.4%)** | **19 (86.4%)** | **10 (58.8%)** |  |
| **AgAb cat:** |  |  |  | **0.857** |
| **Equivocal** | **2 (5.13%)** | **1 (4.55%)** | **1 (5.88%)** |  |
| **Non Reactive** | **10 (25.6%)** | **5 (22.7%)** | **5 (29.4%)** |  |
| **Reactive** | **27 (69.2%)** | **16 (72.7%)** | **11 (64.7%)** |  |
| **Blip** |  |  |  | **1.000** |
| **No** | **29 (72.5%)** | **17 (73.9%)** | **12 (70.6%)** |  |
| **Yes** | **11 (27.5%)** | **6 (26.1%)** | **5 (29.4%)** |  |
| **WB score** | **1.00 [0.50;2.00]** | **1.50 [0.50;2.88]** | **1.00 [0.50;1.50]** | **0.359** |
| **Ultrasensitive VL** | **2.37 [1.05;2.72]** | **2.63 [1.30;4.03]** | **1.29 [1.05;2.63]** | **0.068** |
| **HIVP24** | **0.00 [0.00;0.00]** | **0.00 [0.00;0.00]** | **0.00 [0.00;0.00]** | **0.569** |
| **sPD-L1** | **63.4 [53.4;76.4]** | **71.4 [54.7;86.2]** | **62.9 [49.7;70.8]** | **0.159** |
| **IL10** | **1.37 [1.03;1.66]** | **1.35 [0.97;1.85]** | **1.38 [1.11;1.58]** | **0.692** |
| **IL6** | **0.88 [0.66;1.28]** | **0.93 [0.68;1.69]** | **0.86 [0.50;1.09]** | **0.167** |
| **TNFa** | **3.04 [2.33;3.74]** | **3.01 [2.31;3.67]** | **3.06 [2.48;3.81]** | **0.753** |
| **sPD1** | **389 [252;576]** | **279 [231;510]** | **444 [353;607]** | **0.050** |
| **IP10** | **2.89 [1.98;4.64]** | **2.81 [2.04;4.83]** | **3.26 [1.96;4.14]** | **0.924** |
| **MCP1** | **3.97 [2.64;4.82]** | **3.97 [2.80;4.44]** | **3.46 [2.51;4.87]** | **0.865** |
| **sVCAM-1** | **28842 [16731;42854]** | **41673 [35052;54487]** | **20127 [13632;30530]** | **0.012** |
