# Supplementary material for: Low unspliced cell-associated HIV RNA in early treated adolescents living with HIV on long suppressive ART
Source: Front Immunol. 2024 Feb 20;15:1334236. doi: 10.3389/fimmu.2024.1334236 (PMC10912947; doi:10.3389/fimmu.2024.1334236)
Supplement: Supplementary Table 2 — Immunological and virological markers of CARMA participants with detectable or undetectable unspliced CA-RNA. [file Table_2.docx]

**Supplementary Table S2: Immunological and virological markers of CARMA participants with detectable or undetectable unspliced CA-RNA**

|  | **Total** | **unspliced CA-RNA >0cp/mL** | **unspliced CA-RNA 0cp/mL** | **p-value** |
| --- | --- | --- | --- | --- |
|  | ***N=40*** | ***N=19*** | ***N=21*** |  |
| **Gender:** |  |  |  | **1.000** |
| **Male** | **13 (32.5%)** | **6 (31.6%)** | **7 (33.3%)** |  |
| **Female** | **27 (67.5%)** | **13 (68.4%)** | **14 (66.7%)** |  |
| **Age at ART (m)** | **4.08 [0.25;6.23]** | **4.59 [3.95;10.3]** | **2.66 [0.00;5.28]** | **0.008** |
| **Age at HIV diagnosis** | **4.17 [2.19;6.32]** | **4.20 [3.33;6.14]** | **4.13 [1.51;6.26]** | **0.560** |
| **Baseline Viral load (log10 cp/mL)** | **5.60 [4.98;5.93]** | **5.69 [5.04;5.80]** | **5.52 [5.00;6.01]** | **0.674** |
| **Time to suppression (m)** | **4.69 [2.52;6.26]** | **4.13 [3.46;6.33]** | **4.85 [2.26;5.51]** | **0.957** |
| **DNA amounts** | **48.3 [6.65;113]** | **87.9 [39.6;211]** | **17.9 [0.80;50.9]** | **0.009** |
| **Baseline CD4 count** | **1515 [637;2235]** | **1510 [726;1803]** | **1621 [564;3009]** | **0.570** |
| **Baseline % CD4** | **31.0 [18.0;38.0]** | **30.0 [21.0;36.0]** | **33.5 [18.0;44.2]** | **0.614** |
| **Baseline % CD8** | **32.0 [25.0;40.0]** | **36.0 [25.0;44.0]** | **29.0 [24.0;36.2]** | **0.329** |
| **AntiCMV IGG cat:** |  |  |  | **0.273** |
| **Negative** | **10 (25.6%)** | **3 (15.8%)** | **7 (35.0%)** |  |
| **Positive** | **29 (74.4%)** | **16 (84.2%)** | **13 (65.0%)** |  |
| **AgAb cat:** |  |  |  | **0.306** |
| **Equivocal** | **2 (5.13%)** | **0 (0.00%)** | **2 (10.0%)** |  |
| **Non Reactive** | **10 (25.6%)** | **4 (21.1%)** | **6 (30.0%)** |  |
| **Reactive** | **27 (69.2%)** | **15 (78.9%)** | **12 (60.0%)** |  |
| **Blip** |  |  |  | **0.366** |
| **No** | **29 (72.5%)** | **12 (63.2%)** | **17 (81.0%)** |  |
| **Yes** | **11 (27.5%)** | **7 (36.8%)** | **4 (19.0%)** |  |
| **WB score** | **1.00 [0.50;2.00]** | **2.00 [0.75;3.00]** | **0.50 [0.00;1.50]** | **0.044** |
| **Ultrasensitive VL** | **2.37 [1.05;2.72]** | **2.63 [1.25;2.82]** | **1.87 [1.05;2.63]** | **0.499** |
| **HIVP24** | **0.00 [0.00;0.00]** | **0.00 [0.00;0.00]** | **0.00 [0.00;0.00]** | **0.928** |
| **sPD-L1** | **63.4 [53.4;76.4]** | **67.4 [55.4;81.7]** | **62.9 [52.2;73.7]** | **0.456** |
| **IL10** | **1.37 [1.03;1.66]** | **1.32 [0.99;1.85]** | **1.38 [1.09;1.59]** | **0.579** |
| **IL6** | **0.88 [0.66;1.28]** | **1.09 [0.70;1.60]** | **0.77 [0.50;1.13]** | **0.107** |
| **TNFa** | **3.04 [2.33;3.74]** | **2.91 [2.19;3.67]** | **3.25 [2.48;3.94]** | **0.424** |
| **sPD1** | **389 [252;576]** | **279 [240;434]** | **444 [311;607]** | **0.081** |
| **IP10** | **2.89 [1.98;4.64]** | **2.73 [1.97;4.30]** | **3.26 [2.09;4.74]** | **0.507** |
| **MCP1** | **3.97 [2.64;4.82]** | **3.97 [2.55;4.77]** | **3.88 [2.77;4.77]** | **0.910** |
| **sVCAM-1** | **28842 [16731;42854]** | **29778 [20717;43673]** | **24742 [14197;41787]** | **0.586** |
