# Supplementary material for: Low unspliced cell-associated HIV RNA in early treated adolescents living with HIV on long suppressive ART
Source: Front Immunol. 2024 Feb 20;15:1334236. doi: 10.3389/fimmu.2024.1334236 (PMC10912947; doi:10.3389/fimmu.2024.1334236)
Supplement: Supplementary Table 5 — Association of clinical markers and cytokines/inflammation markers with total CA-RNA. [file Table_5.docx]

**Supplementary Table S5: Association of clinical markers and cytokines/inflammation markers with total CA-RNA**

|  | **Association with detectable total CA-RNA** | | | | **Association with Undetectable total CA-RNA** | | | |
| --- | --- | --- | --- | --- | --- | --- | --- | --- |
|  | **Univariable** | | **Multivariable** | | **Univariable** | | **Multivariable** | |
|  | **IRR**  **[CI 95%]** | ***p*-value** | **IRR**  **[CI 95%]** | ***p*-value** | **OR**  **[CI 95%]** | ***p*-value** | **OR**  **[CI 95%]** | ***p*-value** |
| **US-VL** | **0.94**  **[0.88-1.03]** | **0.161** | **1.11**  **[0.86-1.66] ^†^** | **0.267** | **1.39 [1.03-2.44]** | **0.170** | **1.58**  **[1.05-3.59] ^†^** | **0.179** |
| **p24** *(x 1000)* | **0.82**  **[0.55-1.28]** | **0.065** | **0.81**  **[0.56-0.87]** ^†^ | **0.043** | **0.89 [0.70-1.03]** | **0.200** | **0.87**  **[0.69-1.03] ^†^** | **0.150** |
| **Western Blot** | **1.57**  **[1.0-2.85]** | **0.177** | **0.52**  **[0.24-1.20] ^†^** | **0.075** | **1.29 [0.82-2.18]** | **0.290** | **1.52**  **[0.76-3.66] ^†^** | **0.280** |
| **TNFa** | **0.59**  **[0.14-2.42]** | **0.321** | **1.76**  **[0.45-6.11] ^†^** | **0.239** | **0.92 [0.46-1.82]** | **0.810** | **1.04**  **[0.50-2.23] ^†^** | **0.908** |
| **MCP-1** | **0.67**  **[0.38-1.0]** | **0.016** | **0.67**  **[0.45-0.99]** ^†^ | **0.013** | **0.94 [0.74-1.16]** | **0.540** | **0.97**  **[0.74-1.26] ^†^** | **0.756** |
| **IL-6** | **1.04**  **[0.84-2.12]** | **0.840** | **0.9**  **[0.59-2.22] ^†^** | **0.617** | **2.16 [1.01-8.48]** | **0.200** | **2.41**  **[0.98-11.50] ^†^** | **0.400** |
| **IL-10** | **0.90**  **[0.50-2.67]** | **0.756** | **1.12**  **[0.5-2.6] ^†^** | **0.678** | **1.35 [0.83-3.68]** | **0.390** | **1.35**  **[0.81-4.15] ^†^** | **0.071** |
| **sPD-L1** | **1.03**  **[0.99-1.1]** | **0.085** | **1.07**  **[1.03-1.12]** ^†^ | **0.0001** | **1.03 [1.0-1.08]** | **0.080** | **1.05**  **[1.0-1.11] ^†^** | **0.190** |
| **sPD-1** | **0.99**  **[0.99-1.001]** | **0.115** | **0.99**  **[0.99-1.0]** ^†^ | **0.028** | **1.0 [0.99-1.0]** | **0.080** | **1.0**  **[0.99-1.0]** ^†^ | **0.050** |

**† Adjusted by age at ART, baseline % CD4, and baseline viral load**
