# Supplementary material for: Low unspliced cell-associated HIV RNA in early treated adolescents living with HIV on long suppressive ART
Source: Front Immunol. 2024 Feb 20;15:1334236. doi: 10.3389/fimmu.2024.1334236 (PMC10912947; doi:10.3389/fimmu.2024.1334236)
Supplement: Supplementary Table 6 — Association of clinical markers and cytokines/inflammation markers with unspliced CA-RNA. [file Table_6.docx]

**Supplementary Table S6: Association of clinical markers and cytokines/inflammation markers with unspliced CA-RNA**

|  | **Association with detectable unspliced CA-RNA** | | | | **Association with Undetectable unspliced CA-RNA** | | | |
| --- | --- | --- | --- | --- | --- | --- | --- | --- |
|  | **Univariable** | | **Multivariable** | | **Univariable** | | **Multivariable** | |
|  | **IRR**  **[CI 95%]** | ***p*-value** | **IRR**  **[CI 95%]** | ***p*-value** | **OR**  **[CI 95%]** | ***p*-value** | **OR**  **[CI 95%]** | ***p*-value** |
| **US-VL** | **0.90**  **[0.78-1.11]** | **0.210** | **0.79**  **[0.54-1.04] ^†^** | **0.063** | **0.98**  **[0.90-1.04]** | **0.610** | **0.99**  **[0.90-1.05] ^†^** | **0.780** |
| **p24** *(x 1000)* | **1.14**  **[0.85-1.57]** | **0.208** | **0.79**  **[0.53-1.05] ^†^** | **0.061** | **0.93**  **[0.75-1.08]** | **0.40** | **0.89**  **[0.66-1.06] ^†^** | **0.290** |
| **Western Blot** | **2.03**  **[1.003-4.91]** | **0.024** | **0.54**  **[0.27-1.12] ^θ^** | **0.094** | **1.64**  **[1.01-2.91]** | **0.060** | **1.04**  **[0.52-2.09] ^†^** | **0.917** |
| **TNFa** | **0.46**  **[0.11-2.79]** | **0.123** | **1.83**  **[0.62-5.79] ^†^** | **0.161** | **0.77**  **[0.38-1.50]** | **0.450** | **0.79**  **[0.36-1.66] ^†^** | **0.531** |
| **MCP-1** | **0.73**  **[0.53-1.01]** | **0.056** | **0.96**  **[0.71-1.36] ^†^** | **0.781** | **0.95**  **[0.75-1.17]** | **0.640** | **0.94**  **[0.70-1.22] ^†^** | **0.650** |
| **IL-6** | **1.02**  **[0.82-2.24]** | **0.877** | **0.86**  **[0.58-1.68] ^†^** | **0.342** | **1.91**  **[1.02-6.30]** | **0.210** | **2.28**  **[0.98-9.01] ^†^** | **0.578** |
| **IL-10** | **1.15**  **[0.5-4.41]** | **0.620** | **0.74**  **[0.32-2.06] ^†^** | **0.227** | **0.9**  **[0.5-1.36]** | **0.630** | **0.85**  **[0.36-1.38] ^†^** | **0.177** |
| **sPD-L1** | **0.99**  **[0.99-1.0]** | **0.529** | **1.004**  **[0.97-1.04]^†^** | **0.792** | **1.01**  **[0.98-1.04]** | **0.630** | **1.01**  **[0.98-1.05] ^†^** | **0.490** |
| **sPD-1** | **0.99**  **[0.99-1.01]** | **0.112** | **1.0**  **[0.99-1.004] ^†^** | **0.809** | **1.0**  **[0.99-1.00]** | **0.130** | **1.0**  **[0.99-1.0] ^†^** | **0.060** |

**† Adjusted by age at ART, baseline % CD4, and baseline viral load; θ Adjusted by age at ART**
